# Male and female sex hormones in primary headaches

**DOI:** 10.1186/s10194-018-0922-7

**Published:** 2018-11-29

**Authors:** Zoë Delaruelle, Tatiana A. Ivanova, Sabrina Khan, Andrea Negro, Raffaele Ornello, Bianca Raffaelli, Alberto Terrin, Dimos D. Mitsikostas, Uwe Reuter, on behalf of the European Headache Federation School of Advanced Studies (EHF-SAS)

**Affiliations:** 1 Department of Neurology, University Hospital Ghent, Corneel Heymanslaan 10, 9000 Ghent, Belgium; 2 First Moscow State Medical University, Moscow, Russia; 3 Danish Headache Center, Glostrup Hospital, Copenhagen, Denmark; 4 Dipartimento di Medicina Clinica e Molecolare, Universita degli Studi di Roma La Sapienza, Rome, Italy; 5 Department of Neurology, University of La’Aquila, L’Aquila, Italy; 6 Departmentt of Neurology, Charité Universitätsmedizin Berlin, Berlin, Germany; 7 Department of Neurosciences, Headache Center, University of Padua, Padua, Italy; 8 Neurology Department, Aeginition Hospital, National and Kapodistrian University of Athens, Athens, Greece; 9 Charite Universitatsmedizin Berlin, Berlin, Germany

**Keywords:** Primary headache, Migraine, Tension-type headache, Cluster headache, Sex hormones, Estrogen, Testosterone, Gender

## Abstract

**Background:**

The three primary headaches, tension-type headache, migraine and cluster headache, occur in both genders, but all seem to have a sex-specific prevalence. These gender differences suggest that both male and female sex hormones could have an influence on the course of primary headaches. This review aims to summarise the most relevant and recent literature on this topic.

**Methods:**

Two independent reviewers searched PUBMED in a systematic manner. Search strings were composed using the terms LH, FSH, progesteron*, estrogen*, DHEA*, prolactin, testosterone, androgen*, headach*, migrain*, “tension type” or cluster. A timeframe was set limiting the search to articles published in the last 20 years, after January 1st 1997.

**Results:**

Migraine tends to follow a classic temporal pattern throughout a woman’s life corresponding to the fluctuation of estrogen in the different reproductive stages. The estrogen withdrawal hypothesis forms the basis for most of the assumptions made on this behalf. The role of other hormones as well as the importance of sex hormones in other primary headaches is far less studied.

**Conclusion:**

The available literature mainly covers the role of sex hormones in migraine in women. Detailed studies especially in the elderly of both sexes and in cluster headache and tension-type headache are warranted to fully elucidate the role of these hormones in all primary headaches.

## Introduction

The primary headaches covered in this review are tension-type headache (TTH), migraine and cluster headache (CH). All three entities occur in both men and women, yet display a sex-specific prevalence. These gender differences suggest that both male and female sex hormones could have an influence on the course of primary headaches.

TTH has a female preponderance, and is 1.5 times more frequent in women than in men [1]. CH, on the other hand, appears to have a higher incidence in men, specifically during young adulthood and middle age. Later in life the prevalence of CH evens out between the sexes [2]. Within the group of primary headaches the role of sex hormones has been studied most profoundly in migraine. Prepubertal children have a 3-10% prevalence of migraine without any gender difference [3, 4]. With onset of puberty and its associated hormonal changes, migraine becomes 2–3 times more common in women than in men, suggesting that migraine is influenced by the fluctuating hormonal status through menarche, menstruation, pregnancy, menopause, as well as the use of oral contraceptives and hormonal replacement therapy (HRT) [1, 3, 5–8].

In contrast, the course of migraine throughout the lifespan of men appears relatively stable, further pointing to the unique role of female sex hormones in the migraine phenotype [1]. Here, we summarise relevant literature of the last 20 years covering the influence of female and male sex hormones on primary headaches.

## Search strategy and selection criteria

Two independent reviewers conducted a search on PubMed, using their own search string, composed of terms like LH, FSH, Progesteron*, estrogen*, DHEA*, Prolactin, Testosterone, androgen*AND Headach* OR Migrain* OR “Tension type” OR Cluster. This general search was performed on December 7th, 2017. In light of the large amount of published work on the topic and considering the evolution of the diagnostic criteria over time, the first search was conducted respecting a timeframe of 20 years, covering articles published after January 1st 1997. The initial screening was performed based on eligibility of title and abstract. Exclusion criteria included non-availability of abstract, animal studies, and articles in any language other than English. Original studies, published in full, constitute the core of this review. Other quoted references include systematic reviews, case reports, meta-analysis, Cochrane reviews, letters, lectures and comments. Any relevant publications cited in the eligible articles were also included. Differences between reviewers were resolved by careful discussion.

## Results

### Women

#### Childhood and adolescence

Almost 60% of girls and 50% of boys suffer from headache at some time during childhood and adolescence, with the prevalence increasing significantly during adolescence in girls, whereas it remains stable for boys [9]. The incidence of migraine is similar in both sexes until the age of 9 (2.5% of girls and 2.4% of boys) and then diverges to the disadvantage of girls [6]. Teenagers who suffer from headache are at greater risk of having headache in adulthood [9].

It is known that during puberty, sexual steroid hormones affect neural circuits and cause permanent changes in important brain areas such as the hypothalamus and the insula [4]. Onset of migraine frequently occurs around the time of menarche, as cyclic hormonal changes begin. Early menarche appears to be a risk factor for the development of migraine [6, 10]. Notably, the first menstrual cycles are often anovulatory and in general ovulation occurs one or two years later. In the USA, the average age of menarche is 12.8 years, but this may vary geographically. Migraine with aura has an incidence peak between ages 12 to 13, while migraine without aura typically presents a few years later. Thus, migraine without aura may be associated with the establishment of a regular ovulatory menstrual cycle [7]. Headaches are reported in 53% of adolescent girls at the onset of menses. Pubertal development and age seem to modulate the effect of ovarian hormones on migraine. In fact, high urinary levels of pregnandiol glucuronide, a metabolite of progesterone, are associated with a higher migraine frequency in girls before menarche, but with a lower frequency after menarche [11]. Hershey et al. identified specific genomic patterns in girls suffering from menstrual migraine, suggesting a genetic predisposition for the development of this condition during adolescence [12].

TTH shows a similar, increasing trend in girls by the time of menarche. The incidence ratio between boys and girls changes from 1.3:1 during childhood to 1:1.2 after menarche [13].

It is noteworthy to mention, that pathological changes in sexual hormones can cause a secondary headache. For instance, hyperprolactinemia manifests in up to 45% of childhood cases with headache as a first symptom [14–16].

#### Adulthood

##### Migraine

Women have a 3.25-fold higher risk of suffering from migraine than men [17]. A prevalence peak is reached in women between the ages of 35 and 45, with 25-30% of the general female population being affected, in comparison to only 8% of the general male population [18]. Female migraine patients also report a significant higher burden of disease and greater use of analgesic compared to men [6, 13].

In terms of deciphering the pathophysiological mechanism of the preponderance of migraine in women, neuroimaging studies have revealed sex-specific activation patterns, with an increased activation of the insula and precuneus in women. These regions are involved in pain, sensation and affective processing [19]. Sex hormones can cross the blood-brain barrier passively and are at least partially responsible for these sex differences [18]. Most available literature focuses on the effects of estrogen, while the role of progesterone has been less thoroughly investigated.

The relationship between estrogen and migraine is complex, involving modulation by genomic and non-genomic effects [20, 21]. Obese women appear to have more than a twofold risk of episodic and chronic migraine, probably due to the pathological estrogen production in adipose tissue [22, 23]. Substantial evidence points to the serotonergic system as a key player in migraine pathogenesis [7]. Estrogen modulates serotonergic neurotransmission, by increasing the expression of the tryptophan hydroxylase and decreasing the expression of the serotonin reuptake transporter [7, 24, 25]. Estrogen also activates the endogenous opioidergic system, which has an analgesic effect on persistent, inflammatory pain [26]. Furthermore, estrogen induces vascular changes by modulating vasodilation and suppressing vascular inflammatory responses [6, 27, 28].

The levels of calcitonin gene-related peptide (CGRP), a neuropeptide with a key role in migraine pathophysiology, are higher in women of reproductive age than in men. Cyclic hormonal fluctuations influence CGRP release and consequently the trigeminovascular system [29]. While studies have reported a positive relationship between CGRP and estrogen levels, newer studies suggest an inverse relationship between the two [24].

Experimental studies suggest progesterone to play a protective role, by reducing nociception in the trigeminovascular system, inhibiting neurogenic edema, and histamine secretion from mast cells and decreasing prostaglandin production [7, 24, 30, 31].

Multiple studies have examined the association between polymorphisms in estrogen or progesterone receptor genes and migraine risk, with inconclusive findings [32–37]. In their meta-analysis, Schürks et al. and Li et al. concluded that exon 4 325C > G and exon 8 594G > A polymorphisms are risk factors for migraine, while the often examined PROGINS variant in the progesterone receptor gene did not seem to play a significant role in the Caucasian population [38, 39]. On the contrary, Joshi et al. found a protective role of the PROGINS polymorphism in an Indian population [40].

Prolactin could also play a modulatory role in migraine. Parashar et al. found higher prolactin levels in migraineurs compared to controls [41]. An association between high prolactin levels and migraine chronification has been proposed by Cavestro et al. [42], where Peres et al. detected decreased nocturnal prolactin peaks in chronic migraine patients [43].

There are a few reports suggesting that testosterone can play a role in migraine in women [44, 45]. In one case report, the 5α reductase inhibitor finasteride was administered to a young woman with migraine and led to an almost complete remission [45]. The mechanism of action of testosterone on migraine pathophysiology is still unknown, but may involve modulation of cerebral blood flow, serotonergic tone, and susceptibility to cortical spreading depression [44].

##### Menstrual migraine

The probability of migraine to occur during the perimenstrual period is twice as high compared to any other moment of the menstrual cycle [46]. Almost half of female migraine patients report an association between headache and their menstrual cycle [17]. Depending on whether migraine occurs exclusively during the perimenstrual period or also at other times, the International Headache Society (IHS) distinguishes a pure menstrual migraine from a menstrually-related migraine (Table 1). Migraine associated with menstruation is mostly of the type without aura [21].

**Table 1:** IHS classification (ICHD-3) for pure menstrual and menstrually-related migraine

Pure menstrual migraine	Menstrually-related migraine
A. Attacks, in a menstruating woman, fulfilling criteria for migraine without aura	A. Attacks, in a menstruating woman, fulfilling criteria for migraine without aura
B. Attacks occur exclusively on day 1 ± 2 (i.e, days − 2 to + 3)^a^ of menstruation^b^ in at least two out of three menstrual cycles and at no other times of the cycle	B. Attacks occur on day 1 ± 2 (i.e, days − 2 to + 3)^a^ of menstruation^b^ in at least two out of three menstrual cycles and additionally at other times of the cycle

^a^The first day of menstruation is day 1 and the preceding day is day − 1; there is no day 0

^b^For the purposes of this classification, menstruation is considered to be endometrial bleeding resulting from either the normal menstrual cycle or from the withdrawal of exogenous progestogens, as in the case of combined oral contraceptives and cyclical hormone replacement therapy

Pure menstrual migraine and menstrually-related migraine have an overall prevalence of respectively 1% and 7% in the general population [47]. Data from specialized headache clinics suggest that perimenstrual attacks are more severe, long-lasting and difficult to treat with abortive anti-migraine medication [48]. However, these results could not be confirmed in the general population [49]. Menstrual migraine appears to limit work and social activities more frequently than common migraine and is often associated with a dysphoric mood [17].

The “Estrogen withdrawal hypothesis”, developed by Somerville and colleagues in 1972, postulates that attacks of menstrual migraine are triggered by the decrease in estrogen levels preceding menstruation [21]. A drop in estrogen may cause an increased sensitivity to prostaglandins and a release of neuropeptides such as CGRP, substance P and neurokinins which could result in neurogenic inflammation [17]. This physiological response provokes alterations in the microvasculature of the dura mater, changes in calcium and magnesium concentrations, and an imbalance in serotonin and dopamine concentrations [17, 21, 50]. Estrogen withdrawal might lead to an increased oxidative stress in the cells [51]. To confirm this hypothesis, intramuscular injections of estrogen were administered before menstruation and thereby postponing migraine attacks [52, 53]. On the contrary, progesterone injections only led to postpone menses, but not migraine [52, 54].

More recent studies confirm that an estrogen drop can trigger migraine, especially if this drop is preceded by a phase of high estrogen levels, as in the luteal phase of the menstrual cycle, and if the magnitude of the decrease is greater than 10 μg [55, 56]. Interestingly, women with migraine seem to have a faster drop in estrogen levels than non-migraineurs [57].

Welch et al. tried to explain estrogen effects on menstrual migraine with a “mismatch theory”. Under normal circumstances, genomic effects of estrogen can counterbalance non-genomic mediated membrane excitability. In low estrogen states, this inhibiting genomic effect does not suffice, and migraine attacks occur more frequently [58, 59].

In one retrospective study with 85 female patients with menstrual migraine, 35.3% reported migraine headache onset by the end of menstruation, which is days after the estrogen drop. The authors hypothesize that this type of migraine headache is not related to hormonal changes but most probably to transient anemia due to blood loss [56].

Hormonal treatment of menstrual migraine, like perimenstrual application of estrogen gel or a transdermal estradiol patch, can lead to less frequent, shorter and less intensive attacks [46, 47, 52, 60]. Attacks may recur after discontinuation of hormonal treatment [17]. Following the estrogen withdrawal hypothesis, eliminating estrogen cycling appears to be a useful strategy for long-term prophylaxis of menstrual migraine. Therefore, continuous combined contraceptive therapy regimes, containing both estrogen and progesterone, can be considered. However, there is currently no evidence that hormonal therapy is more effective than non-hormonal pharmacological treatment strategies. Hormonal therapy is particularly recommended if other indications like acne or hirsutism exist. Contraindications should be ruled out [17, 53]. Alternatively, progesterone-only contraceptives can be considered. A significant reduction in migraine intensity and frequency is reported [17, 61–63]. As progesterone has no experimental effect on cortical spreading depression, progesterone-only contraception is hypothesized to be a safer choice for women with aura [62, 64], but no clinical evidence has confirmed this theory. The selective estrogen receptor modulator Tamoxifen might also be beneficial in women with menstrual migraine. However, its use is not generally recommended due to possible and in part serious side effects [65]. Some studies suggest that phytoestrogens like soy isoflavone, dong quai or black cohosh could have a beneficial effect on migraine [17]. Martin et al. examined the efficacy of the gonadotropin-releasing hormone antagonist goserelin as a prophylactic therapy. Goserelin alone did not affect migraine headache frequency. Some benefit was obtained when combined with 100 μg estradiol [66]. Glaser et al. demonstrated that continuous testosterone therapy through a subcutaneous implant for 3 months led to headache improvement in 92% of migraine patients [44].

##### Migraine with aura

The female dominance is also seen in migraine with aura. In prevalence studies performed after 1988 it reaches a prevalence of 1.2-3.7% in men and 2.6-10.8% in women [67]. In contrast to menstrual migraine, migraine with aura occurs more frequently with high estrogen levels [68]. Estrogen seems to change cortical susceptibility and contributes to the development of cortical spreading depression. The amplitude of the spreading depression depends on the estrogen level [69]. The threshold for cortical excitability and subsequent cortical spreading depression is lowered through several genomic and non-genomic mechanisms, including upregulation of NMDA receptors, downregulation of GABA neurons and modulation of axonal plasticity [4, 69, 70].

##### Exogenous hormone-induced headache

In the Western world, almost one third of women of reproductive age use oral contraception [55]. The IHS identifies two headache entities related to the use of hormonal contraceptives: exogenous hormone-induced headache and estrogen-withdrawal headache (Table 2).

**Table 2:** IHS classification (ICHD-3) for exogenous hormone-induced headache and estrogen-withdrawal headache

Exogenous hormone-induced headache	Estrogen-withdrawal headache
A. Headache or migraine fulfilling criteria C and D	A. Headache or migraine fulfilling criteria C and D
B. Regular use of exogenous hormones	B. Daily use of exogenous estrogen for ≥3 weeks, which has been interrupted
C. Headache or migraine develops or markedly worsens within 3 months of commencing exogenous hormones	C. Headache or migraine develops within 5 days after last use of estrogen
D. Headache or migraine resolves or reverts to its previous pattern within 3 months after total discontinuation of exogenous hormones	D. Headache or migraine resolves within 3 days of its onset

Headache is one of the most common side effects of hormonal therapies [71]. For instance Tamoxifen, mentioned above as a possible treatment for menstrual migraine, can also cause headache. The onset of hormone-induced headache is typically within the first months of use [72]. Combined contraception remedies (oral pill, transdermal patch, vaginal ring) appear to be associated with both migraine and non-migraine headaches [73]. The effect in migraine patients is variable. One out of two female migraine patients report no change of the headache pattern, 15% experience an improvement, while 28% report worsening [74]. A negative effect occurs more often in migraine with aura [72]. Headaches most frequently occur in the “pill-free” week [53]. The neuronal nociceptive sensitivity is increased in this week and the probability of getting a headache is 20% higher [74, 75]. Higher age (> 35 years) and a positive family history for migraine are risk factors [76, 77].

Possible contraceptive strategies to reduce headache include extended-cycle combined hormonal contraception, progesterone-only contraception or new generation hormones like estradiol valerate/dienogest [17, 62, 78, 79]. Eliminating the pill-free week is associated with improvement of headache, pelvic pain and quality of life [55].

In progestin-only methods (oral pill, subdermal implant, depot-injection, levonorgestrel-releasing intrauterine system) headache is a common complaint at the beginning of therapy but classically improves after a few months. There is no known association between progestin-only methods and the worsening of migraine [74]. On the contrary, frequency and intensity of migraine can significantly improve with this type of contraception. Ten percent of patients discontinue treatment due to side effects, particularly spotting [80, 81].

Migraine with aura is associated with a twofold risk of major cardiovascular events, like ischemic stroke. This risk is directly proportional to aura frequency [55]. In the meta-analysis of Schürks et al. a relative stroke risk of 1.73 (95% CI 1.31-2.29) was found for any type of migraine. The relative risk of stroke in women suffering from migraine with aura is 2.08 (95% CI 1.3-3.31). The relative risk of cardiovascular deaths in women with migraine is 1.60 (95% CI 1.72-2.43) [82]. Older combined hormonal therapies with high dosed estrogen (50–150 μg) are associated with a 4.4-fold risk of stroke in migraine patients, in particular in migraine with aura and should not be used anymore. The modern low-estrogen contraceptives (< 25 μg) seem much safer [55, 56]. The 2017 consensus statement from the European Headache Federation and the European Society of Contraception and Reproductive Health recommends against the use of combined hormonal contraceptives in women with migraine with aura seeking hormonal contraception. They postulate a strong recommendation to prefer non-hormonal (condoms, copper-bearing intrauterine device, permanent methods) or progestogen-only alternatives. The same strategy is preferred in women with migraine without aura who have additional cardiovascular risk factors, like smoking, arterial hypertension, previous history of a trombo-embolic event. When there are no such risk factors, combined hormonal contraceptives are considered a possible contraceptive option with monitoring of migraine frequency and characteristics in women without aura. Other medical conditions like polycystic ovary syndrome or endometriosis can influence the risk/benefit profile and have an impact on the preferred type of contraception [83].

##### Tension-type headache

The impact of hormones on TTH is less frequently studied. Like migraine, TTH occurs more often in women than in men and some studies have suggested an increase during hormonal changes such as menses or pregnancy. Menstruation can be an aggravating factor in 40-60% of patients [13]. There is no evidence that TTH is influenced by hormonal contraception [77].

##### Cluster headache

The hypothalamus is thought to be involved in CH pathophysiology based on its periodic time locked occurrence. Sex hormones appear to modulate hypothalamic activity and could be effective as a treatment for therapy refractory CH [84]. Both male and female cluster patients show low testosterone levels and testosterone supplementation could have a positive effect on headache attacks [2]. In the first studies from the early 1990s, testosteron supplementation did not prove effective, but more recent data show a good response in a subgroup of cluster patients [84]. Clomifen is a selective estrogen modulator, primarily used for ovulatory stimulation in women. In men, it leads to an increase in luteinizing and follicle stimulating hormones (LH, FSH) and subsequently to higher testosterone levels. Furthermore, in animal model, it reduces prostaglandine production [85]. In a case-series of 7 patients with chronic cluster headache and 8 patients with episodic cluster headache, Clomifen led to pain freedom after 15 days on average [84].

Evidence of dysregulation of the hypothalamus-hypophysial axis in trigeminal autonomic cephalgias could be derived from a case with high nocturnal prolactin levels in a female patient suffering from short, unilateral, neuralgiform headache with conjunctival injection and tearing (SUNCT) [86].

##### Other headache types

Pituitary diseases are often associated with secondary headaches. Especially in female patients with prolactinoma, migraine-like headaches or worsening of a known migraine are reported. Mainly mechanic aspects such as compression of pain sensitive structures play a role in the development of headache, but probably increased hormonal secretion has an impact as well [87]. Prolactin is involved in regulation of neuronal excitability and neurotransmission efficacy [88]. Headache is commonly localized on the same side of the tumor and gets better after treatment with dopamine agonists [89, 90].

#### Perimenopause

Perimenopause is a period of decrease in reproductive capability in middle-aged women. During this period the growth and development of ovarian follicles stops and the pattern of estrogen and progesterone production changes. Signs of perimenopause include irregular menses and periodic amenorrhea starting several years before menopause, also called the menopausal transition. The average age of onset is 40 to 55 years and the average duration is 4 years, but in some women perimenopause can last from several months up to 10 years [91].

The Stages of Reproductive Aging Workshop developed a classification for staging reproductive aging dividing a women’s life into three stages based on the menstrual cycle: premenopausal (or reproductive), perimenopausal (or menopausal transition) and menopausal (or postmenopause) phase. There are two phases in the menopausal transition: the early phase, characterized by a variable cycle length (≥ 7 days), and a late amenorrhea phase. Postmenopause can also be divided into two stages. An early stage that lasts 5 to 8 years, characterized by amenorrhea length more than 1 year, low estrogen levels and high FSH level. The late stage is characterized by stable low levels of ovarian hormones [92].

Perimenopause is characterized by fluctuations in both estrogen and progesterone levels. Due to these constant rapid changes in concentrations of ovarian hormones 60-70% of perimenopausal women experience symptoms such as headaches, flushing, mood swings, depression, decreased libido and sleep disturbance [91]. The decrease of estrogen in the late luteal phase leads to low blood serum estrogen and progesterone levels and promotes prostaglandins release by the uterus influencing the menstrual cycle. This estrogen withdrawal becomes more frequent and longer and can have a secondary impact on headache patterns [46, 93].

##### Migraine

Studies show that migraine prevalence in menopause is lower compared to the perimenopausal period. Menopausal transition seems to negatively impact migraine frequency [94, 95]. As perimenopause and menopause consist of several phases, each with a unique hormonal pattern, they all have a different effect on migraine. Another important factor is whether the menopause is naturally or artificially induced and whether HRT is used [92].

Fluctuation in the estrogen level is a known migraine trigger. The hormonal alterations during perimenopause can provoke migraine attacks in 50% of women with menstrual migraine and menstrual related migraine. Rather stable levels of estrogen are replaced by a more fluctuating pattern with periods of rapid decline in estrogen concentration, the so called estrogen withdrawal [95–97]. The amount of estrogen withdrawal episodes is correlated to headache attack frequency in women with menstrual migraine in “early” perimenopause. Likewise women can experience an increase in menstruation frequency and in some cases an increase in vaginal bleeding duration and severity [98]. This is related to an increase in uterine prostaglandins, which also influences central pain mechanisms and the trigeminovascular system provoking menstrual migraine attacks [99, 100]. Another potential mechanism that can increase menstrual migraine attack frequency is iron deficiency caused by menstrual bleeding [101]. Depression, chronic pain syndrome and sleep disturbance can be other symptoms related to perimenopause, which in turn can lead to a secondary increase in migraine [102].

Women suffering from the premenstrual syndrome were shown to experience more migraine attacks in late perimenopause. The attack frequency declines in the menopausal period. The premenstrual syndrome seems a predictor of migraine attack frequency increase for women entering menopause. These women are considered to have high sensitivity to hormonal fluctuations and liability to moderately severe climacteric symptoms, which in turn can have an impact on migraine [92].

##### Migraine and hormonal replacement therapy (HRT)

HRT is used to ease climax symptoms during menopausal transition. It seems to have a significant influence on migraine course. Studies confirm the correlation between the use of HRT, both oral and topical, and migraine [103, 104]. Oral high dosed estrogen can provoke new onset migraine with aura or worsening of pre-existent migraine with aura. Nappi et al. concluded that migraine deteriorated in women using oral estradiol plus medroxyprogesterone acetate. The course of the disease did not change with a transdermal patch [105]. A few years later MacGregor et al. showed that transdermal patches with estrogen can be effective in decreasing migraine attack frequency in perimenopausal and postmenopausal women, supposedly more effectively than oral contraceptives [106]. Gels and patches based on estradiol seem preferable over oral variants as constant blood hormones levels are maintained stable. They should be taken continuously without omission to prevent rapid changes in estrogen blood levels, a known trigger for migraine [105, 107]. These fluctuations in estrogen concentration have a more significant impact on migraine than progesterone levels. Nand et al. studied three groups of patients treated with different doses of progesterone combined with estrogen and revealed that changes in progesterone levels have no influence on migraine course [92].

HRT containing low doses of natural estrogens are linked to an insignificant risk of thromboembolism, in contrast to the above mentioned combined oral contraception. Nevertheless HRT should be stopped immediately in case of a new onset migraine with aura, a clear increase in frequency or worsening of migraine with aura, transitory ischemic attack or other vascular pathology [108].

##### Migraine and surgical menopause

Natural menopause seems to reduce migraine frequency, in contrast to surgically induced menopause [5]. Neri et al. studied a group of postmenopausal women [109]. Improvement of migraine was seen in two thirds of cases compared to the premenopausal period. At the same time no reduction in days with TTH was observed. In women, who underwent ovariectomy the course of migraine worsened in the majority of women (67%). Thirtythree percent reported migraine improvement. In women with natural menopause 67% reported improvement in migraine course, in 24% of patients no change was observed and 9% reported worsening [109]. There is still a debate on possible migraine worsening in women who undergo procedures such as hysterectomy, dilation and curettage or cesarean section. Arumugam and Parthasarathy found a positive correlation between these procedures and the prevalence of migraine in women [110]. Oldenhave et al. compared a group of 986 hysterectomized women and 5636 women without hysterectomy with one or both ovaries preserved. The amount of days without migraine in the group without hysterectomy was less compared to the hysterectomy group. This data confirms the importance of presence or absence of the uterus on migraine frequency in menopausal women [92].

##### Tension-type headache

The most common risk factors for TTH are considered to be stress, fatigue and sleep disturbance. During perimenopause these symptoms can exacerbate and trigger TTH. But TTH also seems to have a correlation with reproductive hormone levels [111]. In some women menstruation can trigger TTH and also pregnancy and menopause can influence the course of TTH [93, 111]. In retrospective evaluations 38% to 46% of women reported an increase of headache rate during menstruation [112, 113]. Arjona et al. even tried to identify “menstrual TTH” and “menstrual related TTH” based on ICHD-2 criteria for pure menstrual migraine and menstrually-related migraine. These terms were not included into the ICHD [114]. Women in the perimenopause reported their headaches to have new characteristics and prevalence of TTH seems rather high [115]. The prevalence of TTH in postmenopausal women is reported to be higher than in premenopausal women [116].

##### Cluster headache

According to the literature the course of CH in women is biphasic. The first peak of onset is seen around the age of 20 and the second at age 50 to 60. The majority of female cluster patients experience their first attack during menopause [116, 117]. The role of estrogen in CH and the reason for CH onset in these women remain unclear. Estrogen receptors are seen in the trigeminal ganglion and in sensory neurons which makes them susceptible to rapid changes in estrogen level [118]. In menopause the reduced level of estrogen is assumed to provoke CH, while the higher estrogen level in the premenopausal phase can have a protective effect [119]. However, based on the available literature, there is no clear evidence on the relationship between CH and hormonal changes in women [120, 121].

In 2006 van Vliet et al. published a large retrospective study in which data from more than 200 women with CH were analyzed using questionnaires. Among women with CH 9% reported more intense CH attacks during menstruation, while frequency didn’t change. Eighty-six percent of women were using lifelong oral contraceptives in this trial. Initiation of oral contraceptives was associated with an increase of days with headache in 12% of participants. In 4% of the cases headache frequency was reduced. Out of 111 pregnant women with episodic CH 26 (23%) women reported “expected” CH attacks not to occur. After childbirth 8 of them experienced CH attacks in the first month. Nineteen patients (17%) had attacks during pregnancy and 11 of them did not report any changes in attack frequency or intensity [120].

#### Elderly

In the elderly, headache is less frequent compared to younger patients. Headache disorders are mostly primary, but the relative frequency of secondary headache is higher in the elderly [122]. In a random population sample, the prevalence of headache in women and men aged 55 to 74 years is approximately 66% and 53%, respectively, compared to 92% and 74%, respectively, in their younger counterparts between the ages of 21 to 34 years. The prevalence further declines in patients aged over 75 to 55% for women and 22% in men [123]. In a population survey, the prevalence of frequent headache in elderly women was 20% and 10% in elderly men [124]. Another survey showed a 3-month prevalence of headache among patients aged more than 66 years of 40.6% in men and 49.7% in women [125]. In summary, all studies show that headache is more prevalent in women compared to men at all ages, even among the elderly. Hormonal factors take account for the sex-specific difference in headache prevalence. However, literature data about the relationship between headache and hormonal activity in elderly women are scarce. Only the relationship between migraine and estrogen has been extensively studied in older women, possibly because of the high prevalence of migraine and its sensitivity to hormonal fluctuations.

Up to 51.9% of elderly patients referred for specialist consultation report onset of headache after 65 years of age [126]. Some primary headache disorders, and mostly hypnic headache, have the tendency to start after the age of 50, in contrast to most primary headache disorders, which usually start at a younger age. However, migraine still accounts for 0.5% of all new-onset headache disorders after the age of 65 [127, 128]. The low estrogen level in elderly women may explain why onset of migraine in this age group is uncommon. Migraine with onset at older age affects women and men equally, while in younger age groups women outnumber men [129].

##### Migraine

As mentioned above, the “estrogen withdrawal hypothesis” attributes migraine episodes to the fluctuation of estrogen levels throughout women’s reproductive events. After menopause, women’s serum levels of estradiol drop. A lower frequency and severity of migraine episodes is expected because of the stable low serum levels of estrogen. Migraine prevalence declines after menopause compared to the fertile period. However, the prevalence of migraine after the menopause is still 10 to 29% across studies [5].

Interestingly, the decreased burden of migraine after the menopause is more evident in population-based studies when compared with those performed in headache clinics or menopause clinics [94, 109, 115, 130–134]. This can be explained by a possible selection bias towards more severe forms of migraine in clinic-based studies as compared to population-based studies [5]. Menopause has a different and variable effect on migraine with or without aura [8]. In a population-based study, the burden of migraine without aura decreased after menopause while that of the variant with aura remained stable [130]. In a headache clinic-based study migraine without aura remained unchanged or even worsened in the majority of patients possibly because of the above mentioned selection bias of clinic-based studies [135]. Collectively, these data suggest that migraine without aura improves more frequently after menopause compared to migraine with aura. This can be a possible consequence of migraine without aura being more sensitive to female sex hormones [5]. However, the available studies might have failed to show any change in the frequency of migraine with aura after the menopause because of low statistical power [136]. When migraine with aura does not subside with age, characteristics may change, with increasing occurrence of aura without headache. These auras constitute a difficult differential diagnosis with transient ischemic attacks [137, 138]. An aura is generated by cortical spreading depression while migraine pain has been linked to the neurovascular system. Elderly subjects may exhibit an intact cortical spreading depression phenomenon, while the propensity to neurovascular inflammation declines [139]. It is likely that those changes can be a consequence of the postmenopausal estrogen drop. However, to the best of our knowledge, this has not been proven yet.

Together with female sex hormones, male sex hormones might have an influence on the course of headache disorders among elderly women. Only one case-control study assessed the levels of androstenedione and testosterone in the serum of postmenopausal women with and without migraine and found no differences in the levels of these hormones when comparing women with and without migraine [140].

In conclusion, the postmenopausal drop of estrogen might be beneficial for elderly women with migraine. However, the proportion of women experiencing migraine in menopause is still relevant.

##### Tension-type headache

The effect of menopause on TTH is less clear than the corresponding effect on migraine. One population-based study addressing the topic found that the frequency of TTH decreased less than that of migraine after menopause. However, that same study pointed out that fluctuations of sex hormone levels during the life cycle might influence TTH as well as migraine [131].

##### Hormonal therapy

Hormonal manipulation in elderly women cannot be considered for migraine prevention at this time. HRT is contraindicated from 10 years after menopause or in women aged 60 years or older due to its potential cardiovascular side effects [141]. No other hormonal therapy has been attempted in the prevention of migraine in elderly women. Clomiphene citrate has been used to treat chronic cluster headache and refractory primary SUNCT in single cases of elderly males [142, 143]. Clomiphene has a direct effect on hypothalamic estrogen receptors and estrogen modulates hypothalamic orexin expression. Hypothalamic estrogen receptors co-localize to orexin neurons. Therefore, clomiphene might upregulate orexin A levels, which in turn inhibits the trigeminal nucleus caudalis activity and secondarily suppresses the trigemino-autonomic reflex, preventing hypothalamic-driven headache [142]. These results are promising in considering hormonal therapies as prevention for headache disorders in elderly women. However, there are no studies to date.

### Males

#### Migraine

Migraine is notoriously known to be two to three times more prevalent in women than in men. Migraine is characterized by its fluctuating nature, where periods of remission are interspersed by relapse, with men more likely to have longer periods of remission compared to women. This female dominance of migraine suggests that factors increasing female vulnerability and/or protecting males deserve greater focus in migraine pathophysiology [144]. Interestingly, a study has shown that male-to-female transsexuals who use antiandrogens to suppress male sex characteristics and estrogens to induce female sex characteristics have migraine rates similar to genetic females, further adding to the notion that gender-specific hormones play a role in migraine prevalence. The authors suggest that this similarity in migraine prevalence could include structural differences in the transsexual brain or that migraine headache is part of the female gender role [145].

Animal models of migraine have attempted to investigate the gender specific difference in migraine prevalence. In an animal model of familial hemiplegic migraine type 1 (FHM1), it has been shown that orchiectomy increases susceptibility to cortical spreading depression, a response partially reversed with testosterone replacement [146]. Also, female FHM1 mutant mice were more susceptible to cortical spreading depression than males [146–148].

Another explanation for increased prevalence of migraine in women could be attributed to inherent differences in pain perception and processing. The fundamental subjectivity of pain perception complicates quantification of pain, yet it is generally accepted that women and men experience pain differently due to both biological and psychosocial traits [144]. Clinical studies are often not designed to decipher gender-specific difference [149].

#### Cluster headache

In contrast to migraine, cluster headache has traditionally been considered a male disease [150]. While the characteristic physical attributes of cluster headache patients could point to high testosterone levels, the exact opposite has been shown to be true [151]. Low testosterone levels in patients with episodic and chronic cluster headaches were first noted in the 1970ies and later reproduced [152–154]. Another study found low testosterone levels in the episodic but not chronic cluster headache, a difference attributed by the authors to the disruption of REM sleep [154].

The role of testosterone in cluster headache was further studied by Stillman et al. in their investigation of laboratory findings of 7 male and 2 female patients with treatment refractory cluster headache. Results of all 9 patients demonstrated low serum testosterone levels. After supplementation with either pure testosterone in the male patients or combination testosterone/estrogen therapy in the female patients, pain freedom was achieved for the first 24 h. Four male chronic cluster patients achieved headache remission. The authors concluded that abnormal testosterone levels in patients with episodic or chronic cluster headaches refractory to maximal medical management may be predictive of therapeutic response to testosterone replacement therapy [2].

## Discussion

Reviewing recent literature, it becomes evident that most experimental data on the causal relationship between sex hormones and primary headaches covers women suffering from migraine in the reproductive or perimenopausal phase of their life. Particularly the effect of estrogen has been studied and has been found to be of considerable value in the pathogenesis of migraine. The estrogen withdrawal hypothesis plays a central role here, but it is assumed that this is only part of the mechanism. Some therapeutic strategies have been developed based on this knowledge. Continuous combined contraceptive therapy regimes can be considered as a treatment for menstrual migraine. However, there is currently no evidence to support the superiority of hormonal therapy over non-hormonal pharmacological treatment strategies. When using hormonal therapies in migraine patients, whether it is as a contraceptive or as a treatment, potential cardiovascular risks should be considered when deciding which type of hormones to use.

For the other primary headaches and more so ever for headaches in male patients, the role of sex hormones is vague. Is there more to know? It seems plausible that trying to uncover the effects of sex hormones on the other primary headaches may offer new insights in pathophysiological mechanisms. The more we know on this matter, the more targeted possible new therapies can be.

## Conclusion

All three primary headaches, migraine, TTH, and CH, occur in both genders, but with a sex-specific prevalence. Also, headache patterns display a temporal evolution that correlates to the hormonal shifts of a life cycle. Collectively, these findings suggest that both male and female sex hormones could play an important role in the pathophysiology of primary headaches. Reviewing the available literature on this matter, we can conclude that especially the role of estrogen in female migraine patients has been well-studied. Detailed studies especially in the elderly of both sexes, in CH, and TTH are warranted in order to clearly elucidate the role of sex hormones in not just migraine, but all primary headaches.

## Abbreviations

CGRP: Calcitonin gene-related peptide
CH: Cluster headache
FHM1: Familial hemiplegic migraine type 1
FSH: Follicle stimulating hormone
GABA: Gamma-aminobutyric acid
HRT: Hormonal replacement therapy
ICHD: International Classification of Headache Disorders
IHS: International Headache Society
LH: Luteinizing hormone
NMDA: N-methyl-D-aspartate
SUNCT: Short, unilateral, neuralgiform headache with conjunctival injection and tearing
TTH: Tension-type headache

## References

[1] Cairns BE, Gazerani P (2009). Sex-related differences in pain. Maturitas.

[2] Stillman MJ (2006). Testosterone replacement therapy for treatment refractory cluster headache. Headache.

[3] Silberstein SD (2000). Sex hormones and headache. Rev Neurol (Paris).

[4] Borsook D, Erpelding N, Lebel A (2014). Sex and the migraine brain. Neurobiol Dis.

[5] Ripa P, Ornello R, Degan D (2015). Migraine in menopausal women: a systematic review. Int J Womens Health.

[6] Pavlovic JM, Akcali D, Bolay H (2017). Sex-related influences in migraine. J Neurosci Res.

[7] Martin VT, Behbehani M (2006). Ovarian hormones and migraine headache: understanding mechanisms and pathogenesis--part I. Headache.

[8] Martin VT, Behbehani M (2006). Ovarian hormones and migraine headache: understanding mechanisms and pathogenesis--part 2. Headache.

[9] Bjorling EA, Singh N (2017). Exploring Temporal Patterns of Stress in Adolescent Girls with Headache. Stress Health.

[10] Aegidius KL, Zwart JA, Hagen K (2011). Increased headache prevalence in female adolescents and adult women with early menarche. The Head-HUNT Studies. Eur J Neurol.

[11] Martin VT, Allen JR, Houle TT et al (2017) Ovarian hormones, age and pubertal development and their association with days of headache onset in girls with migraine: An observational cohort study. Cephalalgia. 10.1177/0333102417706980 10.1177/0333102417706980 28474986

[12] Hershey A, Horn P, Kabbouche M (2012). Genomic expression patterns in menstrual-related migraine in adolescents. Headache.

[13] Marcus DA (2001). Sex hormones and chronic headache in women. Expert Opin Pharmacother.

[14] Eren E, Yapici S, Cakir ED (2011). Clinical course of hyperprolactinemia in children and adolescents: a review of 21 cases. J Clin Res Pediatr Endocrinol.

[15] Catli G, Abaci A, Altincik A (2012). Hyperprolactinemia in children: clinical features and long-term results. J Pediatr Endocrinol Metab.

[16] Catli G, Abaci A, Bober E (2013). Clinical and diagnostic characteristics of hyperprolactinemia in childhood and adolescence. J Pediatr Endocrinol Metab.

[17] Allais G, Chiarle G, Sinigaglia S (2018). Menstrual migraine: a review of current and developing pharmacotherapies for women. Expert Opin Pharmacother.

[18] Martin VT, Lipton RB (2008). Epidemiology and biology of menstrual migraine. Headache.

[19] Vincent K, Tracey I (2010). Sex hormones and pain: the evidence from functional imaging. Curr Pain Headache Rep.

[20] Craft RM (2007) Modulation of pain by estrogens. Pain 132(Suppl 1):S3–S12 10.1016/j.pain.2007.09.028 17951003

[21] Tassorelli C, Greco R, Allena M (2012). Transdermal hormonal therapy in perimenstrual migraine: why, when and how?. Curr Pain Headache Rep.

[22] Horev A, Wirguin I, Lantsberg L (2005). A high incidence of migraine with aura among morbidly obese women. Headache.

[23] Fava A, Pirritano D, Consoli D (2014). Chronic migraine in women is associated with insulin resistance: a cross-sectional study. Eur J Neurol.

[24] Gupta S, Mccarson KE, Welch KM (2011). Mechanisms of pain modulation by sex hormones in migraine. Headache.

[25] Zacur HA (2006). Hormonal changes throughout life in women. Headache.

[26] Warnock JK, Cohen LJ, Blumenthal H (2017). Hormone-Related Migraine Headaches and Mood Disorders: Treatment with Estrogen Stabilization. Pharmacotherapy.

[27] Krause DN, Duckles SP, Pelligrino DA (2006). Influence of sex steroid hormones on cerebrovascular function. J Appl Physiol (1985).

[28] Miller VM, Duckles SP (2008). Vascular actions of estrogens: functional implications. Pharmacol Rev.

[29] Labastida-Ramirez A, Rubio-Beltran E, Villalon CM et al (2017) Gender aspects of CGRP in migraine. Cephalalgia. 10.1177/0333102417739584 10.1177/0333102417739584 PMC6402050 29082826

[30] Gruber CJ, Huber JC (2003). Differential effects of progestins on the brain. Maturitas.

[31] Vasiadi M, Kempuraj D, Boucher W (2006). Progesterone inhibits mast cell secretion. Int J Immunopathol Pharmacol.

[32] Colson NJ, Lea RA, Quinlan S (2004). The estrogen receptor 1 G594A polymorphism is associated with migraine susceptibility in two independent case/control groups. Neurogenetics.

[33] Colson NJ, Lea RA, Quinlan S (2006). No role for estrogen receptor 1 gene intron 1 Pvu II and exon 4 C325G polymorphisms in migraine susceptibility. BMC Med Genet.

[34] Lee H, Sininger L, Jen JC (2007). Association of progesterone receptor with migraine-associated vertigo. Neurogenetics.

[35] Mehrotra S, Gupta S, Chan KY (2008). Current and prospective pharmacological targets in relation to antimigraine action. Naunyn Schmiedeberg's Arch Pharmacol.

[36] Rodriguez-Acevedo AJ, Maher BH, Lea RA (2013). Association of oestrogen-receptor gene (ESR1) polymorphisms with migraine in the large Norfolk Island pedigree. Cephalalgia.

[37] Sutherland HG, Champion M, Plays A (2017). Investigation of polymorphisms in genes involved in estrogen metabolism in menstrual migraine. Gene.

[38] Schurks M, Rist PM, Kurth T (2010). Sex hormone receptor gene polymorphisms and migraine: a systematic review and meta-analysis. Cephalalgia.

[39] Li L, Liu R, Dong Z (2015). Impact of ESR1 Gene Polymorphisms on Migraine Susceptibility: A Meta-Analysis. Medicine (Baltimore).

[40] Joshi G, Pradhan S, Mittal B (2010). Role of the oestrogen receptor (ESR1 PvuII and ESR1 325 C->G) and progesterone receptor (PROGINS) polymorphisms in genetic susceptibility to migraine in a North Indian population. Cephalalgia.

[41] Parashar R, Bhalla P, Rai NK (2014). Migraine: is it related to hormonal disturbances or stress?. Int J Womens Health.

[42] Cavestro C, Rosatello A, Marino MP (2006). High prolactin levels as a worsening factor for migraine. J Headache Pain.

[43] Peres MF, Sanchez Del Rio M, Seabra ML (2001). Hypothalamic involvement in chronic migraine. J Neurol Neurosurg Psychiatry.

[44] Glaser R, Dimitrakakis C, Trimble N (2012). Testosterone pellet implants and migraine headaches: a pilot study. Maturitas.

[45] Check JH, Cohen R (2013). Dihydrotestosterone may contribute to the development of migraine headaches. Clin Exp Obstet Gynecol.

[46] Macgregor EA, Frith A, Ellis J (2006). Incidence of migraine relative to menstrual cycle phases of rising and falling estrogen. Neurology.

[47] Maasumi K, Tepper SJ, Kriegler JS (2017). Menstrual Migraine and Treatment Options: Review. Headache.

[48] Martin VT, Wernke S, Mandell K (2005). Defining the relationship between ovarian hormones and migraine headache. Headache.

[49] Newman LC (2007). Understanding the causes and prevention of menstrual migraine: the role of estrogen. Headache.

[50] Glinskii OV, Huxley VH, Glinsky VV (2017). Estrogen-Dependent Changes in Dura Mater Microvasculature Add New Insights to the Pathogenesis of Headache. Front Neurol.

[51] Borkum JM (2016). Migraine Triggers and Oxidative Stress: A Narrative Review and Synthesis. Headache.

[52] Macgregor EA (2009). Estrogen replacement and migraine. Maturitas.

[53] Loder E, Rizzoli P, Golub J (2007). Hormonal management of migraine associated with menses and the menopause: A clinical review. Headache.

[54] Macgregor EA (2004). Oestrogen and attacks of migraine with and without aura. Lancet Neurol.

[55] Calhoun A (2012). Combined Hormonal Contraceptives: Is It Time to Reassess Their Role in Migraine?. Headache.

[56] Calhoun AH, Batur P (2017). Combined hormonal contraceptives and migraine: An update on the evidence. Clev Clin J Med.

[57] Pavlovic JM, Allshouse AA, Santoro NF (2016). Sex hormones in women with and without migraine: Evidence of migraine-specific hormone profiles. Neurology.

[58] Welch KMA, Brandes JL, Berman NEJ (2006). Mismatch in how oestrogen modulates molecular and neuronal function may explain menstrual migraine. Neurol Sci.

[59] Nappi RE, Nappi G (2012). Neuroendocrine aspects of migraine in women. Gynecol Endocrinol.

[60] Almen-Christensson A, Hammar M, Lindh-Astrand L (2011). Prevention of menstrual migraine with perimenstrual transdermal 17-beta-estradiol: a randomized, placebo-controlled, double-blind crossover study. Fertil Steril.

[61] Nappi RE, Sances G, Allais G (2011). Effects of an estrogen-free, desogestrel-containing oral contraceptive in women with migraine with aura: a prospective diary-based pilot study. Contraception.

[62] Nappi RE, Merki-Feld GS, Terreno E et al (2013) Hormonal contraception in women with migraine: is progestogen-only contraception a better choice? J Headache Pain 14 10.1186/1129-2377-14-66 PMC3735427 24456509

[63] Merki-Feld GS, Imthurn B, Dubey R (2017). Improvement of migraine with change from combined hormonal contraceptives to progestin-only contraception with desogestrel: How strong is the effect of taking women off combined contraceptives?. J Obstet Gynaecol.

[64] Allais G, Gabellari IC, De Lorenzo C (2011). Oral contraceptives in migraine therapy. Neurol Sci.

[65] Smitherman TA, Kolivas ED (2010). Resolution of Menstrually Related Migraine Following Aggressive Treatment for Breast Cancer. Headache.

[66] Martin V, Wernke S, Mandell K (2003). Medical oophorectomy with and without estrogen add-back therapy in the prevention of migraine headache. Headache.

[67] Manzoni Gc TP (2003). Epidemiology of migraine. J Headache Pain.

[68] Scharfman HE, Maclusky NJ (2008). Estrogen-growth factor interactions and their contributions to neurological disorders. Headache.

[69] Eikermann-Haerter K, Kudo C, Moskowitz MA (2007). Cortical spreading depression and estrogen. Headache.

[70] Finocchi C, Ferrari M (2011). Female reproductive steroids and neuronal excitability. Neurol Sci.

[71] Lopez-Picado A, Lapuente O, Lete I (2017). Efficacy and side-effects profile of the ethinylestradiol and etonogestrel contraceptive vaginal ring: a systematic review and meta-analysis. Eur J Contracep Repr.

[72] Bousser MG (2004). Estrogens, migraine, and stroke. Stroke.

[73] Aegidius K, Zwart JA, Hagen K (2006). Oral contraceptives and increased headache prevalence - The Head-HUNT Study. Neurology.

[74] Macgregor EA (2013). Contraception and Headache. Headache.

[75] De Icco R, Cucinella L, De Paoli I et al (2016) Modulation of nociceptive threshold by combined hormonal contraceptives in women with oestrogen-withdrawal migraine attacks: a pilot study. J Headache Pain 17 10.1186/s10194-016-0661-6 PMC4972742 27488685

[76] Allais G, Gabellari IC, Airola G (2009). Headache induced by the use of combined oral contraceptives. Neurol Sci.

[77] Loder EW, Buse DC, Golub JR (2005). Headache and combination estrogen-progestin oral contraceptives: integrating evidence, guidelines, and clinical practice. Headache.

[78] Sulak P, Willis S, Kuehl T (2007). Headaches and oral contraceptives: Impact of eliminating the standard 7-day placebo interval. Headache.

[79] Macias G, Merki-Feld GS, Parke S (2013). Effects of a combined oral contraceptive containing oestradiol valerate/dienogest on hormone withdrawal-associated symptoms: Results from the multicentre, randomised, double-blind, active-controlled HARMONY II study. J Obstet Gynaecol.

[80] Allais G, Chiarle G, Bergandi F (2016). The use of progestogen-only pill in migraine patients. Expert Rev Neurother.

[81] Warhurst S, Rofe CJ, Brew BJ et al (2017) Effectiveness of the progestin-only pill for migraine treatment in women: A systematic review and meta-analysis. Cephalalgia. 10.1177/0333102417710636 10.1177/0333102417710636 28554244

[82] Schurks M, Rist PM, Bigal ME (2009). Migraine and cardiovascular disease: systematic review and meta-analysis. BMJ.

[83] Sacco S, Merki-Feld GS, Kl AE (2017). Hormonal contraceptives and risk of ischemic stroke in women with migraine: a consensus statement from the European Headache Federation (EHF) and the European Society of Contraception and Reproductive Health (ESC). J Headache Pain.

[84] Nobre ME, Peres MFP, Moreira PF (2017). Clomiphene treatment may be effective in refractory episodic and chronic cluster headache. Arq Neuro-Psiquiat.

[85] Rozen T (2008). Clomiphene citrate for treatment refractory chronic cluster headache. Headache.

[86] Bosco DLA, Mungari P (2007). SUNCT and high nocturnal prolactin levels: some new unusual characteristics. J Headache Pain.

[87] Kreitschmann-Andermahr I, Siegel S, Weber Carneiro R (2013). Headache and pituitary disease: a systematic review. Clin Endocrinol.

[88] Patil MJ, Henry MA, Akopian AN (2014) Prolactin receptor in regulation of neuronal excitability and channels. Channels 8 10.4161/chan.28946 PMC4203747 24758841

[89] Bosco D, Belfiore A, Fava A (2008). Relationship between high prolactine levels and migraine attacks in patients with macroprolactinoma. J Headache Pain.

[90] Kallestrup MM, Kasch H, Osterby T (2014). Prolactinoma-associated headache and dopamine agonist treatment. Cephalalgia.

[91] Freeman EW, Sammel MD, Lin H (2008). Symptoms in the menopausal transition hormone and behavioral correlates. Obstet Gynecol.

[92] Allais G, Chiarle G, Bergandi F (2015). Migraine in perimenopausal women. Neurol Sci.

[93] Stewart WF, Lipton RB, Chee E (2000). Menstrual cycle and headache in a population sample of migraineurs. Neurology.

[94] Wang SJ, Fuh JL, Lu SR (2003). Migraine prevalence during menopausal transition. Headache.

[95] Martin VT, Pavlovic J, Fanning KM (2016). Perimenopause and Menopause Are Associated With High Frequency Headache in Women With Migraine: Results of the American Migraine Prevalence and Prevention Study. Headache.

[96] Harlow SD, Gass M, Hall JE (2012). Executive Summary of the Stages of Reproductive Aging Workshop+10: Addressing the Unfinished Agenda of Staging Reproductive Aging. J Clin Endocr Metab.

[97] Soules MR, Sherman S, Parrott E (2001). Executive summary - Stages of Reproductive Aging Workshop (STRAW). J Women Health Gen-B.

[98] Van Voorhis BJ, Santoro N, Harlow S (2008). The relationship of bleeding patterns to daily reproductive hormones in women approaching menopause. Obstet Gynecol.

[99] Smith OPM, Jabbour HN, Critchley HOD (2007). Cyclooxygenase enzyme expression and E series prostaglandin receptor signalling are enhanced in heavy menstruation. Hum Reprod.

[100] Martin VT (2008). New Theories in the Pathogenesis of Menstrual Migraine. Curr Pain Headache R.

[101] Vukovic-Cvetkovic V, Plavec D, Lovrencic-Huzjan A (2010). Is Iron Deficiency Anemia Related to Menstrual Migraine? - Post Hoc Analysis of an Observational Study Evaluating Clinical Characteristics of Patients with Menstrual Migraine. Acta Clin Croat.

[102] Odegard SS, Sand T, Engstrom M (2011). The Long-Term Effect of Insomnia on Primary Headaches: A Prospective Population-Based Cohort Study (HUNT-2 and HUNT-3). Headache.

[103] Misakian AL, Langer RD, Bensenor IM (2003). Postmenopausal hormone therapy and migraine headache. J Women's Health.

[104] Aegidius KL, Zwart JA, Hagen K (2007). Hormone replacement therapy and headache prevalence in postmenopausal women. The Head-HUNT study. Eur J Neurol.

[105] Nappi RE, Cagnacci A, Granella F (2001). Course of primary headaches during hormone replacement therapy. Maturitas.

[106] Macgregor EA, Frith A, Ellis J (2006). Prevention of menstrual attacks of migraine - A double-blind placebo-controlled crossover study. Neurology.

[107] Macgregor A (1999). Effects of oral and transdermal estrogen replacement on migraine. Cephalalgia.

[108] Macgregor EA (2012). Perimenopausal migraine in women with vasomotor symptoms. Maturitas.

[109] Neri I, Granella F, Nappi R (1993). Characteristics of Headache at Menopause - a Clinico-Epidemiologic Study. Maturitas.

[110] Arumugam M, Parthasarathy V (2015). Increased incidence of migraine in women correlates with obstetrics and gynaecological surgical procedures. Int J Surg.

[111] Ailani J (2010). Tension-Type Headache and Women: Do Sex Hormones Influence Tension-Type Headache?. Curr Pain Headache R.

[112] Spierings ELH, Ranke AH, Honkoop PC (2001). Precipitating and aggravating factors of migraine versus tension-type headache. Headache.

[113] Zivadinov R, Willheim K, Sepic-Grahovac D (2003). Migraine and tension-type headache in Croatia: a population-based survey of precipitating factors. Cephalalgia.

[114] Arjona A, Rubi-Callejon J, Guardado-Santervas P (2007). Menstrual tension-type headache: Evidence for its existence. Headache.

[115] Oh K, Jung KY, Choi JY (2012). Headaches in Middle-Aged Women during Menopausal Transition: A Headache Clinic-Based Study. Eur Neurol.

[116] Lieba-Samal D, Wober C (2011). Sex Hormones and Primary Headaches Other than Migraine. Curr Pain Headache R.

[117] Ekbom K, Svensson DA, Traff H (2002). Age at onset and sex ratio in cluster headache: observations over three decades. Cephalalgia.

[118] Puri V, Puri S, Svojanovsky SR (2006). Effects of oestrogen on trigeminal ganglia in culture: implications for hormonal effects on migraine. Cephalalgia.

[119] Burger H (2008). The menopausal transition - Endocrinology. J Sex Med.

[120] Van Vliet JA, Favier I, Helmerhorst FM (2006). Cluster headache in women: relation with menstruation, use of oral contraceptives, pregnancy, and menopause. J Neurol Neurosur Ps.

[121] Bahra A, May A, Goadsby PJ (2002). Cluster headache - A prospective clinical study with diagnostic implications. Neurology.

[122] Sharma TL (2018) Common Primary and Secondary Causes of Headache in the Elderly. Headache 10.1111/head.13252 29322494

[123] Waters WE (1974). The pontypridd headache survey. Headache.

[124] Cook NR, Evans DA, Funkenstein HH (1989). Correlates of headache in a population-based cohort of elderly. Arch Neurol.

[125] Boardman HF, Thomas E, Croft PR (2003). Epidemiology of headache in an English district. Cephalalgia.

[126] Ruiz M, Pedraza MI, De La Cruz C (2014). Headache in the elderly: characteristics in a series of 262 patients. Neurologia.

[127] Evers S, Goadsby PJ (2003). Hypnic headache: clinical features, pathophysiology, and treatment. Neurology.

[128] Pascual J, Berciano J (1994). Experience in the diagnosis of headaches that start in elderly people. J Neurol Neurosurg Psychiatry.

[129] Song TJ, Kim YJ, Kim BK (2016). Characteristics of Elderly-Onset (>/=65 years) Headache Diagnosed Using the International Classification of Headache Disorders, Third Edition Beta Version. J Clin Neurol.

[130] Mattsson P (2003). Hormonal factors in migraine: A population-based study of women aged 40 to 74 years. Headache.

[131] Karli N, Baykan B, Ertas M (2012). Impact of sex hormonal changes on tension-type headache and migraine: a cross-sectional population-based survey in 2,600 women. J Headache Pain.

[132] Whitty CW, Hockaday JM (1968). Migraine: a follow-up study of 92 patients. Br Med J.

[133] Cupini LM, Matteis M, Troisi E (1995). Sex-hormone-related events in migrainous females. A clinical comparative study between migraine with aura and migraine without aura. Cephalalgia.

[134] Mueller L (2000). Predictability of exogenous hormone effect on subgroups of migraineurs. Headache.

[135] Granella F, Sances G, Zanferrari C (1993). Migraine without Aura and Reproductive Life Events - a Clinical Epidemiologic-Study in 1300 Women. Headache.

[136] Granella F, Sances G, Pucci E (2000). Migraine with aura and reproductive life events: a case control study. Cephalalgia.

[137] Wijman CA, Wolf PA, Kase CS (1998). Migrainous visual accompaniments are not rare in late life - The Framingham Study. Stroke.

[138] Vongvaivanich K, Lertakyamanee P, Silberstein SD (2015). Late-life migraine accompaniments: A narrative review. Cephalalgia.

[139] Bigal ME, Liberman JN, Lipton RB (2006). Age-dependent prevalence and clinical features of migraine. Neurology.

[140] Mattsson P (2002). Serum levels of androgens and migraine in postmenopausal women. Clin Sci.

[141] Stuenkel CA, Davis SR, Gompel A (2015). Treatment of Symptoms of the Menopause: An Endocrine Society Clinical Practice Guideline. J Clin Endocr Metab.

[142] Rozen TD (2014). Complete alleviation of treatment refractory primary SUNCT syndrome with clomiphene citrate (a medicinal deep brain hypothalamic modulator). Cephalalgia.

[143] Rozen TD (2015). Clomiphene Citrate as a Preventive Treatment for Intractable Chronic Cluster Headache: A Second Reported Case With Long-Term Follow-Up. Headache.

[144] Loewendorf AI, Matynia A, Saribekyan H et al (2016) Roads Less Traveled: Sexual Dimorphism and Mast Cell Contributions to Migraine Pathology. Front Immunol 7 10.3389/fimmu.2016.00140 PMC4836167 27148260

[145] Pringsheim T, Gooren L (2004). Migraine prevalence in male to female transsexuals on hormone therapy. Neurology.

[146] Eikermann-Haerter K, Baum MJ, Ferrari MD (2009). Androgenic Suppression of Spreading Depression in Familial Hemiplegic Migraine Type 1 Mutant Mice. Ann Neurol.

[147] Moskowitz MA, Bolay H, Dalkara T (2004). Deciphering migraine mechanisms: Clues from familial hemiplegic migraine genotypes. Ann Neurol.

[148] Bolay H, Berman NEJ, Akcali D (2011). Sex-Related Differences in Animal Models of Migraine Headache. Headache.

[149] Ramirez-Maestre C, Esteve R (2014). The Role of Sex/Gender in the Experience of Pain: Resilience, Fear, and Acceptance as Central Variables in the Adjustment of Men and Women With Chronic Pain. J Pain.

[150] Rasmussen BK (1995). Epidemiology of Headache. Cephalalgia.

[151] Stillman M (2006). Steroid hormones in cluster headaches. Curr Pain Headache Rep.

[152] Kudrow L (1976). Plasma testosterone levels in cluster headache preliminary results. Headache.

[153] Nelson RF (1978). Testosterone levels in cluster and non-cluster migrainous headache patients. Headache.

[154] Romiti A, Martelletti P, Gallo MF (1983). Low plasma testosterone levels in cluster headache. Cephalalgia.

